# Synthesis, Characterization, and Genotoxic and Cytotoxic In Vitro Evaluation of Ceramic Nanoparticles of Sc Oxide Powders and Aerogels Doped with Europium Ions

**DOI:** 10.3390/gels12070646

**Published:** 2026-07-19

**Authors:** Israel D. Cabrera Rios, Felipe de J. Carrillo Romo, Antonieta García Murillo, Isela Álvarez González, Eduardo Madrigal Bujaidar

**Affiliations:** 1 Instituto Politécnico Nacional CIITEC, Cerrada de Cecati s/n, Azcapotzalco, Santa Catarina, Ciudad de Mexico 02250, Mexicoangarciam@ipn.mx (A.G.M.); 2 Laboratorio de Genética, Escuela Nacional de Ciencias Biológicas, Instituto Politécnico Nacional, Av. Wilfrido Massieu s/n, Zacatenco, Ciudad de Mexico 07738, Mexico

**Keywords:** sol–gel, rare earth, cytokinesis-block micronucleus in vitro, genotoxicity, cytotoxicity

## Abstract

This article reports on the synthesis and characterization of the properties of ceramic powders and aerogels of rare earths using the Sc_2_O_3_:Eu_2_O_3_ system synthesized through the sol–gel method, as well as on the toxicological effects of the cytokinesis-block micronucleus cytome assay (CBMC). A sol–gel variant using epoxide-assisted gelling and supercritical CO_2_ drying was employed to produce the aerogels. In vitro CBMCs were employed to assess the genotoxic and cytotoxic effects of the materials’ dosages and inherent properties. The morphology of the powders and aerogels consisted of agglomerates of irregularly shaped particles. At the same time, structural analysis revealed crystal sizes of 16 and 10 nm, respectively, for the ceramic powders and aerogels, in which microplastic deformations were observed. The cubic crystalline structure of the Sc_2_O_3_:Eu_2_O_3_ system remained unchanged. However, applying CBMC and observing the genotoxic and cytotoxic effects of the nanoparticles revealed that the main genotoxic xenobiotic agent was the aerogel. The primary mode of cellular death was necrosis, suggesting that reactive oxygen species might have been involved in the genotoxic and cytotoxic damage.

## 1. Introduction

The synthesis of engineered materials with particles smaller than 100 nm is advancing the development of new materials with unique properties in both theoretical and applied science, which are performing exceptionally in various fields, including energy development, environmental science, biomedicine, and many others [1,2]. Consequently, various methods have been used to improve the synthesis of new materials, with the sol–gel process being one of the most effective for producing a wide range of products [3]. Notably, aerogels are valued for attractive features such as their highly specific surface area, high porosity, and low thermal conductivity.

Rare earths are prized for their unique properties, their versatility, and their suitability for a great number of high-tech applications, due to their electronic configuration and ability to enhance energy transfer between systems, as well as to act as catalysts or sensitizers. The development of new materials based on rare earths promises applications in several fields of applied science.

This approach is worth exploring in-depth. Aerogels composed of rare-earth elements have a wide range of applications in high-tech industries, and as a result, are in high demand as unique materials that combine macroscopic external and nanoscale internal structures [4,5,6,7]. Nevertheless, the synthesis and characterization of aerogels incorporating a rare-earth matrix remain relatively novel, leaving a significant knowledge gap regarding these materials and their properties, particularly for lanthanide oxide aerogels. Furthermore, the available reports on lanthanide aerogels are confined to the use of alkoxides, chlorides, and nitrates as precursors [8].

Thus, this represents an opportunity to report for the first time on the properties of ceramic powders and aerogels with a rare-earth matrix of scandium oxide, which exhibit exceptional physical and chemical properties, such as high thermal stability, low electrical conductivity, resistance to corrosion, and a cubic crystalline structure suitable for use in advanced high-tech materials [9]. This work marks the beginning of an in-depth study of the properties of ceramic powders and aerogels, including the genotoxic and cytotoxic effects of nanoparticles interacting with biomolecules, and their biocompatibility and bioactivity. Some urgency arises from the increasing, unregulated use of new materials like these and the need to ensure their safety for human and environmental exposure [10,11,12].

The toxicological potential of a nanoparticle is determined by evaluating its behavior when it is in contact with biological systems and by gauging the inherent hazards of the type of particle [13,14,15,16]. Hence, to assess the potential genotoxic and cytotoxic effects of rare-earth nanoparticles currently in use or in the synthesis process, assays that can help deepen our understanding of possible hazards are called for.

Thus, this paper aims to contribute to new knowledge by evaluating two types of particles in cultured human lymphocytes through the use of the cytokinesis-block micronucleus cytome assay (CBMC). This method makes possible the study of several parameters associated with DNA or chromosomal damage and the evaluation of preclinical security, a consideration that attests to its potential use in the biomedical field.

The CBMC enables the detection of genotoxic and cytotoxic events by comparing DNA damage rates among cells, based on the frequency of the formation of micronuclei (MNi), nucleoplasmic bridges (NPBs), and nuclear buds (NBUDs), especially in cells that have completed nuclear division. This can lead to an understanding of the mechanism of action of teratogenic agents and the damage they can cause to DNA or chromosomes at points of mutation, and to an understanding of other forms of genetic damage that result from cellular alterations.

## 2. Results and Discussion

### 2.1. Microstructure

**Figure 1:**
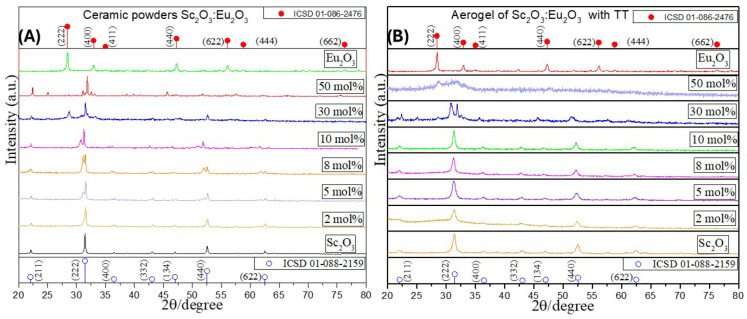
XDR diffractograms of (**A**) the ceramic powders and (**B**) the aerogels of the Sc_2_O_3_:Eu_2_O_3_ system, presenting the crystallographic planes and the formation of twins corresponding to changes in the concentration of europium oxide.

Figure 1 displays the X-ray diffraction (XRD) patterns of the ceramic powders and aerogels used in the microstructure analysis. The system was well crystallized at 700 °C and presents a cubic-centered face structure. The main diffraction peak appears at the (222) plane. For every sample, 2θ = 31.43° of the cubic phase of Sc_2_O_3_, which aligns with the positions of the peaks on the crystallographic chart, at CSD 01-088-2159 and CSD 01-086-2476. The addition of the europium oxide (Eu_2_O_3_) initiated the formation of twins. In Figure 1A, twins formed on all the samples on planes (222) and (440), while in Figure 1B, the twins formed only on the sample with the 30% concentration, on plane (222).

The presence of twins in the samples signifies plastic microdeformation within the crystal structure. These are initially triggered during crystal nucleation, contributing to high yield strength. As deformation progresses, the twins continue to form and grow across grain boundaries [17]. In the ceramic powders in Figure 1A, the addition of the Eu^3+^ ion to the matrix not only enhanced the formation of twins on the principal peak on plane (222) but also produced twins in the secondary plane (440), starting from a concentration of 5%. These also correspond to slips and dislocation movements and the tenacity mechanism that led to the formation of a mixture of phases. The phase mixture was examined by determining the crystallite behavior of the samples. The Scherrer equation (Equation (1)) was used to estimate the sizes at different concentrations:

(1) $$D=\frac{K\lambda }{\beta cos\theta }$$

**Figure 2:**
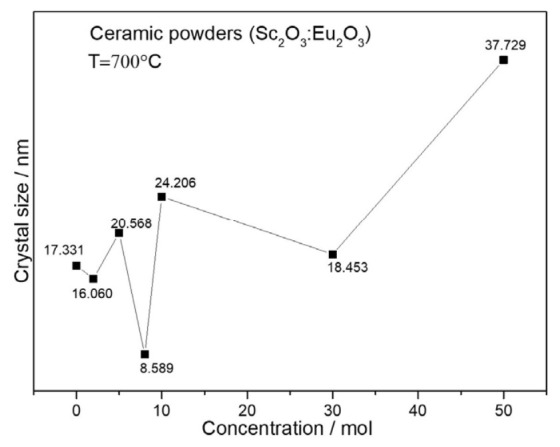
Crystal size versus concentration of ceramic powders of the Sc_2_O_3_:Eu_2_O_3_ system; the behavior of the changing sizes presents mixed phases resulting from the formation of twins due to microplastic deformation at the grain limit, due in turn to compressive stress charges on the crystalline structure.

Figure 2 shows the tendency of crystal growth as the Eu^3+^ ion increases. The tendency, ranging from 17 to 37 nm, is not linear. As noted above, in the diffractograms of the aerogels in Figure 1B, all the samples present crystallographic planes at (211), (222), (440), and (622), located at 22°, 31°, 52°, 62°, respectively, except for the sample with a 30% concentration of Eu^3+^ ions.

**Figure 3:**
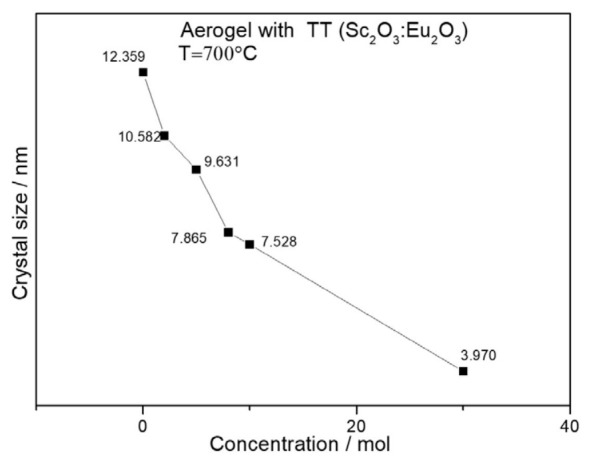
Crystal size versus the concentration of aerogels of the Sc_2_O_3_:Eu_2_O_3_ system; the linear tendency and the crystal sizes can be attributed to supercritical drying and the absence of microplastic deformations.

Here (Figure 3), in contrast, plane (622) disappears and presents the formation of a twin oriented at the 30.78 and 31.91 angles, reflecting structural distortion associated with compressive effects. The sample with a 50% concentration presents semi-crystalline behavior, featuring a peak that started to form on the principal plane (222) of the europium oxide, placed at a 28.62-degree angle, which indicates the beginning of a matrix inversion between the scandium and the europium oxide. Figure 3 shows a graph of the crystal growth tendency of the aerogels. In contrast to the powders, the sizes of the aerogels present a linear, decreasing tendency, ranging from 12 to 3 nm. Distortions at the grain boundaries contribute to the observed plastic deformation in the ceramic powders and the aerogel. For aerogel samples at concentrations of 30% and 50%, the addition of higher concentrations of the europium ion to the matrix of scandium led to an inversion of the matrix, mainly because of the molecular weight, resulting in a high critical resolved shear stress (CRSS). However, compared with the powders, supercritical drying was used on aerogels, as reported by Worsley et al. [14]. This drying process leads to early crystallization that later, with heat treatment, leads to the promotion of the nucleation of the crystal structure, which prevents later slips and vacancies in the atomic arrangement when subjected to heat treatment. In a word, even if the aerogels present the shape and the structure of a gel, they are completely different materials with unique properties. For the powders, the formation of twins can be attributed to compressive loads induced by the drying method or by heat treatment, which can also cause slips along grain boundaries. This results in significant compressive lattice strain, leading to high stress concentrations at grain boundaries. The primary effects include distortions of the crystallographic planes and the formation of partial Shockley dislocations.This results in a highly compressed elastic yield that can exhibit high stress concentrations at grain boundaries, with the main effects being deformations of planes and directions at grain boundaries and partial Shockley dislocations [18,19,20].

### 2.2. Dynamic Light Scattering (DLS) and Zeta Potential

To complement the crystallite size analysis obtained from X-ray diffraction, Dynamic Light Scattering (DLS) measurements were performed to evaluate the hydrodynamic particle size of the samples in suspension. Unlike XRD, which estimates the size of coherent crystalline domains, DLS measures the hydrodynamic diameter of dispersed particles and therefore reflects their aggregation state in the liquid medium.

The Sc_2_O_3_:Eu_2_O_3_ aerogel (2 mol% Eu^3+^) exhibited hydrodynamic diameters ranging from 25.9 to 34.2 nm, with an average value of approximately 30.6 nm. In contrast, the ceramic powder presented larger hydrodynamic diameters, ranging from 54.6 to 180.1 nm. These values are greater than the crystallite sizes estimated by the Scherrer equation, indicating that the nanocrystals in powder form undergo partial aggregation after dispersion in the culture medium.

Zeta potential measurements showed positive surface charges for both materials, with average values of approximately +17.3 mV for the ceramic powders and +12.9 mV for the aerogels. These values indicate moderate colloidal stability, which explains the aggregation behavior observed by DLS. The smaller hydrodynamic diameter of the aerogel is consistent with its highly porous structure generated by the supercritical drying process, which reduces particle packing and limits agglomeration compared with the conventionally dried ceramic powders.

### 2.3. Morphology

**Figure 4:**
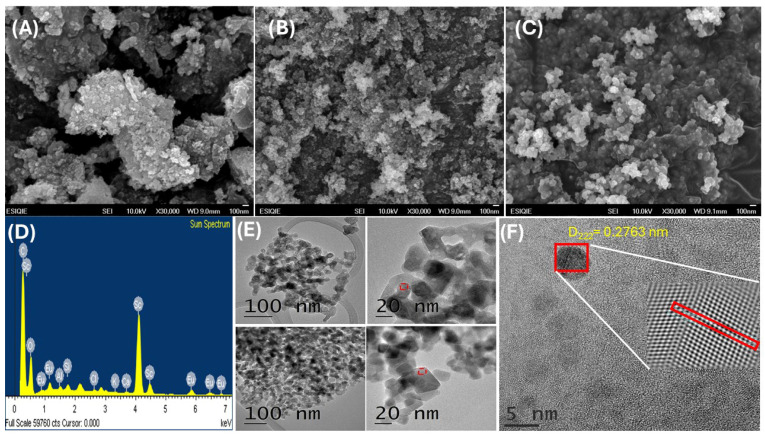
High-resolution SEM images of the particles of Sc_2_O_3_:Eu_2_O_3_ examined at 30,000×: (**A**,**B**) ceramic powders at 100% and 2% concentration; (**C**) aerogels at the 2% concentration; (**D**) elemental map of the samples; (**E**) high-resolution HRTEM images of the ceramic powders at the 2% concentration; (**F**) high-resolution HRTEM images of the aerogels at the 2% concentration examined at 5 nm, showing a morphology similar to that seen in the HRTEM images of the ceramic powders and SEM images.

For the morphological analysis, scanning electron microscopy (SEM) and transmission electron microscopy (TEM) were used to investigate the nanostructures of the powders and aerogels, at 100% and 2% mol of Eu^3+^. Figure 4 presents micrographs at 30,000× magnification. Figure 4A,B display micrographs of ceramic powders at 100% and 2% concentrations, while Figure 4C presents micrographs of the aerogels at 2% concentration. Qualitatively, both materials exhibit clusters of quasi-amorphous, interconnected granular particles with random distributions and sizes. For both the ceramic powders and the aerogels containing Sc_2_O_3_:Eu_2_O_3_ at a 2% concentration, scandium oxide is the predominant crystalline phase, as shown in Figure 4D.

The particle sizes of the samples were 32.43 nm and 35.85 nm at 100% and 2% concentrations, respectively. The aerogel exhibited a particle size of 45.12 nm. Figure 4E displays images from the HRTEM analysis, showing the morphology of a sample of ceramic powders containing 8% europium ion, illustrating the effects of the increasing ion concentration on morphology. The analysis indicated that incorporating higher ion concentrations did not alter the crystal structure or the morphology, as shown in these micrographs.

These analyses are in keeping with the reported dominant planes in the crystal structure, revealing interplanar distances of 2.89, 3.04, and 3.12 nm and aligning with earlier findings. The distances of 2.89 and 3.12 nm align with the main peak on the (222) plane for the oxides Sc and Eu, respectively, as shown in the crystallographic charts, while the 3.04 nm distance is associated with the (220) plane of Sc [9].

Figure 4F displays the HRTEM image of the 2% Eu^3+^ aerogel, showing lattice fringes with an interplanar distance of 2.763 nm, the nearest crystallographic plane (222) aligned with the main peak of Sc_2_O_3_ with a cubic-centered face structure, according to the CSD 01-088-2159 crystallographic chart. This means that the plane’s direction (222) is well integrated, so that molecular accommodation, the layered-structure distribution direction, and anisotropic growth may explain the linear, decreasing tendency in crystal growth, which ranges from 12 to 3 nm. Presented in the XRD before.

**Figure 5:**
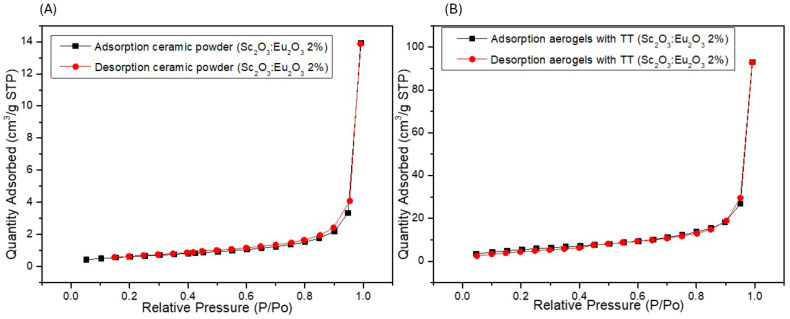
N_2_ adsorption/desorption isotherms of (**A**) the ceramic powders and (**B**) the aerogels of the Sc_2_O_3_:Eu_2_O_3_.

The porous structures of the Sc_2_O_3_ powders and aerogel were measured by N_2_ adsorption/desorption to measure the specific surface area, average pore diameter, and pore volume. Figure 5 shows the N_2_ adsorption/desorption isotherms, all identified as type IV with an H3 hysteresis loop for both ceramic powders and aerogels. When the relative pressure was below 0.8, the isotherm’s gradual rise was due to multilayer adsorption on pore surfaces until the pressure rise resulted in a sharp increase in adsorption [21]. In a word, the isotherms demonstrated the mesopores and macropores on the ceramic powders and the aerogel, shown in Figure 5.

Figure 5A presents the results of the adsorption/desorption of the ceramic powders with a specific surface area of 55.964 m^2^g^−1,^ as the average pore diameter and pore volume of 32.433 nm and 0.719 ccg^−1^, while the N_2_ adsorption/desorption of aerogels on Figure 5B shows a lower specific surface area of 29.246 m^2^g^−1,^ even smaller pore diameters and pore volume of 3.406 nm and 0.144 ccg^−1^, indicated that the capillary condensation within the mesopores of the aerogels are near to micropores order which lead to a unsaturated adsorption, making it difficult to the N_2_ gas to properly permeate those particle sizes, resulting g in low values in terms of specific surface areas, pore diameters and pore volumes.

### 2.4. Cytokinesis-Block Micronucleus Assay (CBMC)

The toxicological assessment of the ceramic powders and aerogels included cytogenetic assays using CBMC, following Fenech’s criteria [22]. Table 1 outlines the concentrations for the ceramic powders (T1) and the aerogels (T2). This study used mitomycin C as the standard xenobiotic to compare healthy cells with those treated with T1 and T2.

This study investigated the in vitro formation of chromosomal damage, possibly induced by nanoparticles, using peripheral blood lymphocytes. This examination could be expanded by assessing additional biomarkers, such as micronuclei, nucleoplasmic bridges, nuclear buds, and necrotic and/or apoptotic cells, thereby providing further insights into cytostasis, genotoxicity, cytotoxicity, and DNA damage and its repair [22,23,24].

In regard to the cytostasis parameter for the toxicology characterization, Equation (2) was used to determine the nuclear division index (NDI):

(2) $$NDI=\frac{1\left(mononucleated\;cells\right)+2\left(binucleated\;cells\right)+3(multinucleated\;cells)}{1000\;total\;de\;cells}$$

**Figure 6:**
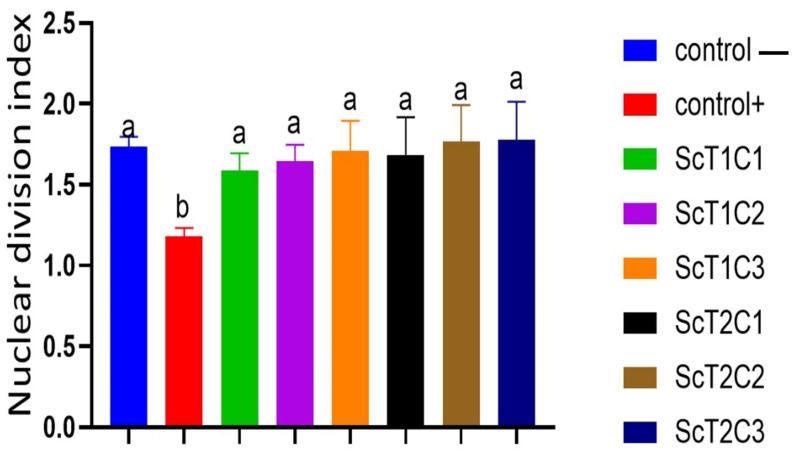
Cytostasis determination of the nanoparticles of Sc_2_O_3_:Eu_2_O_3_, where the ceramic powders (T1) and the aerogels (T2) correspond to the ± 1000 donor cells’ NDIs, based on treatment/concentration. A one-way ANOVA and a Tukey test (*p* ≤ 0.05) were employed to identify statistically significant differences among the samples, which are indicated by the letters “a” and “b”.

Figure 6 demonstrates that the impact of the particles on cellular proliferation was not significant compared to that of the positive control, which contained the antineoplastic agent mitomycin C. Instead, all the cells treated with T1 and T2 presented values comparable to the negative control’s NDI of 1.593, demonstrating that the particles had no negative effect on cell division [25,26].

#### 2.4.1. Genotoxicity

**Figure 7:**
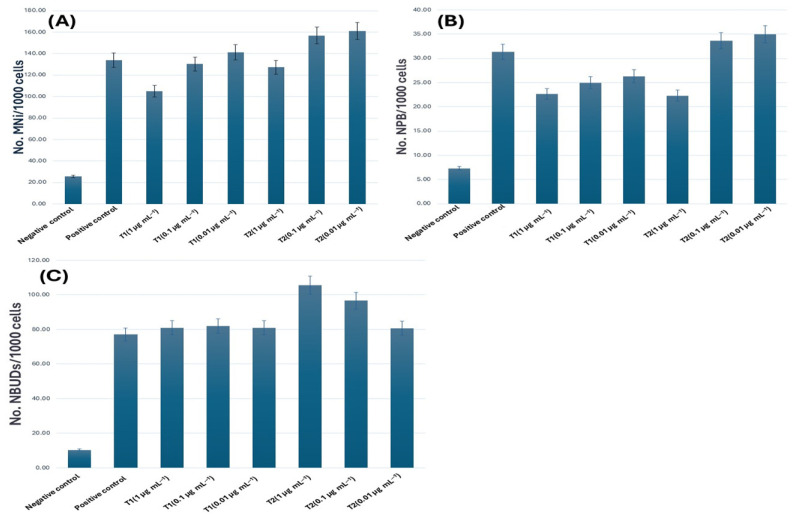
Genotoxic determination parameters: (**A**) micronuclei (MNi); (**B**) nuclei with plasmic bridges (NBPs); (**C**) nuclear blebs (NBUDs) obtained from ± 1000 binucleated donor cells’ treatment/concentration. A one-way ANOVA (*p* ≤ 0.05) was performed.

For the genotoxic parameter, Figure 7A displays the micronuclei (MNi) results, indicating the formation frequency. Initially, the negative control showed a total of 25 cells with MNi, which aligns with the parameters established by Fenech. In contrast, the samples with positive control presented 80 cells with MNi, confirming the genotoxicity of the antineoplastic.

In contrast to the positive control, T1 showed no significant increase in concentrations at 1 and 0.1 µg mL^−1^, compared to 0.01 µg mL^−1^, indicating a 5% increase in formation; for T2, at a concentration of 1 µg mLµg mL^−1^, the behavior was the same as that of T1; however, this differed for the 0.1 and 0.01 µg mL^−1^ concentrations, which increased formation between 17% and 20%. This indicates a genotoxic effect arising from errors produced during cellular replication, such as segregation or chromosomal breaks, which led to the incorrect incorporation of genetic material [22,27].

Figure 7B illustrates the parameters of nucleoplasmic bridges (NPBs). The negative control produced seven out of 1000 cells, in contrast to the positive control, which produced a total of 31 cells. In T1, the generation of NPBs did not surpass that of the positive control at any of its concentrations. Conversely, in the case of T2, the concentrations of 0.1 and 0.01 µg mL^−1^ significantly enhanced the number of cells exhibiting NPBs, exceeding those of the positive control. This suggests that, although the aerogels did not adversely affect the internal mechanisms of cell division, they induced damage during cellular replication. This phenomenon arises from the rupture of dicentric chromosomes and the erroneous reincorporation of telomeres, resulting in a cross-linking of DNA strands, which in turn leads to the formation of bridges that hinder the proper separation of the nuclei [24,28].

This reorganization of chromosomal damage may be connected to repair mechanisms aimed at reintroducing genetic material into the compromised mitotic spindle or the phosphodiester DNA structure.

As regards the last genotoxic biomarker, nucleoplasmic blebs (NBUDs), Figure 7C shows a 5% increase in proliferation for T1 compared to the positive control. In contrast, for T2, a concentration of 1 µg mL^−1^ led to a 38% increase compared to both the positive control and T1. However, at the lower concentrations of 0.1 and 0.01 µg mL^−1^, there was a gradual decrease in the proliferation of nuclear blebs, dropping to 25% at 0.1 µg mL^−1^ and following a downward trend until it reached 5%, similar to the behavior of T1. These results enable an evaluation of the genotoxic damage caused by the nanoparticles by observing gene amplification in nuclei that failed to expel them.

#### 2.4.2. Cytotoxicity

**Figure 8:**
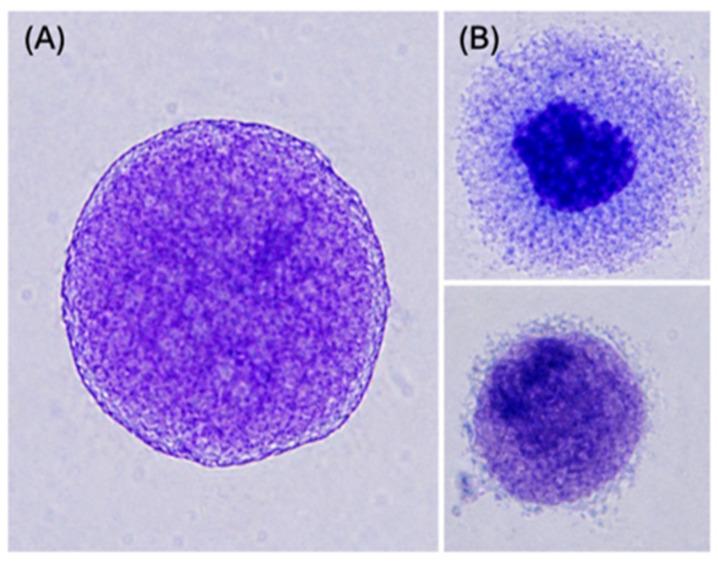
Photomicrographs of (**A**) a necrotic cell vacuolated with the nucleus, which is only marginally intact. and (**B**) cells at various stages of apoptosis treated with scandium particles at concentrations of 1, 0.1, and 0.01 µg mL^−1^, respectively.

The principal mode of cell death, indicated by the cytotoxicity biomarker shown in Figure 8, was necrosis. Starting from the highest concentration to the lowest in T1 and T2, necrotic cells increased seven times compared to the negative control, reaching 54% of the total cells. In the assay, the cells treated with T1 and T2 showed lower values than the positive control, with 11–14% necrotic cell formation. For apoptotic cell formation, T1 showed a significant increase at all concentrations compared to the positive control. T2 at a concentration of 0.1 µg mL^−1^ behaved like the positive control, with minimal differences at the lowest concentrations for both treatments (T1 and T2), which increased from 11% to 20% of the total cell amount compared to the positive control.

Figure 8A shows a photomicrograph of a necrotic cell treated with the scandium particles, showing nuclear condensation, vacuolation, and membrane ruptures. Figure 8B displays a cell containing densely packed apoptotic bodies.

In the case of the T2 samples, the genotoxic behavior for the MNi and NPB parameters is similar, and reducing the concentrations had a greater impact. The effect of the scandium particles is related to the surface principle, which states that the reactivity of particles increases as their size and, consequently, their surface area, is reduced.

A second property of the scandium particles is their greater electrostatic potential, which, combined with their crystalline structure or interatomic forces of attraction, allows direct interactions among them and enables them, once they are internalized by the cell, to bond with biomolecules such as lipids and proteins or with genetic material [29,30,31].

Hence, the materials of which the scandium nanoparticles are composed and their intrinsic properties contribute to their cytotoxicity [29], and these factors impact the cell membrane potential and mitochondrial functions on a molecular level; these may lead to the production of reactive oxygen species (ROS) [31].

Necrosis may be explained by the interactions between the cell and the nanoparticles, which may provoke oxidative stress, disrupting the normal reduction states in the cellular tissues, and cause toxicity by generating peroxides and free radicals, as the principal cause of deteriorative damage in the cell is direct attacks on DNA, proteins, or lipids (including mitochondrial lipids). These damage the cell through inflammation, karyolysis, vacuolation, or lysis, leading to the release of cellular contents and to the death of the cell [11,32,33].

This necrotic process is a form of cell death that occurs uncontrollably and is not influenced by a specific signaling event, whereas the apoptotic process is induced by the activation of caspase proteases and the fixation of intracellular proteins by caspase-9, which activates the apoptosome complex [34].

**Figure 9:**
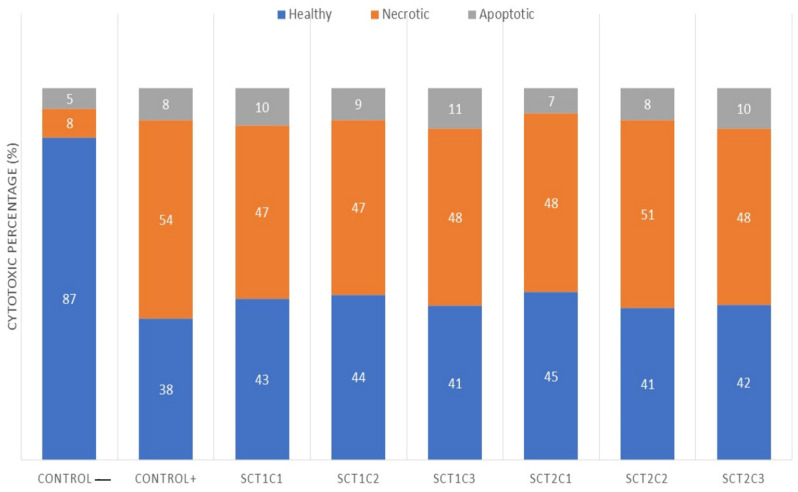
Cytotoxic parameters of necrosis and apoptosis determined from approximately 500 scored cells per treatment/concentration, based on the analysis of 1000 donor cells, expressed as percentages.

In the case of the scandium nanoparticles, the number of healthy cells was lower than the necrotic cells, as is stated in relation to Figure 9, where the exposure of scandium nanoparticles to the cells also targeted cellular tissues that activate pro-apoptotic factors, releasing cytochrome C into the cytosol, initiating the contraction of cellular organelles and the condensation of nuclei, and finally rupturing the packaging of the organelles and fragmenting the cell.

## 3. Conclusions

Engineered nanomaterials like ceramic powders and aerogels of rare earths present a novel opportunity to explore the toxicology of genotoxic and cytotoxic behaviors in cells and the effects these materials can achieve [31,34,35]. These effects relate to properties such as electronegativity charge, which modulates interactions with biomolecules and constituents; particle size (distributions, crystallites); morphology; and even surface area. Such properties lead to increased interactions with cells and facilitate the internalization of particles into cellular membranes where they can compromise vital cell functions [35] and precipitate genotoxic and cytotoxic damage during the replication of genetic material.

The structural analysis in this study revealed an average crystal size of 16 nm for T1 and 10 nm for T2. This difference in size is particularly significant because aerogels are known to be mesoporous materials with a large surface area. The morphological and structural properties observed in this study are therefore highly relevant. In particular, the results obtained for T2 indicate that not only the dose but also the type of nanoparticle is essential when investigating the genotoxicity and cytotoxicity of a material.

Furthermore, it is important to explore how engineered nanomaterials, in their interactions with biomolecules, can evolve and transform to enhance bioavailability by forming biomolecular coronas that significantly influence the reactivity of nanoparticles.

The nanoparticles used in this study show promise for applications in a variety of areas. Investigating the genotoxic and cytotoxic behavior of nanoparticles is essential for gaining deeper insights into how they interact with biomolecules and various media. These insights can contribute to the development of new applications and expand our knowledge across different fields.

## 4. Materials and Methods

### 4.1. Materials

The materials used in this study were scandium oxide (Sc_2_O_3_, 99.9%, Sigma-Aldrich, St. Louis, MO, USA), europium oxide (Eu_2_O_3_, 99.9%, Sigma-Aldrich, St. Louis, MO, USA), propylene oxide (C_3_H_6_O, Sigma-Aldrich, St. Louis, MO, USA, ≥99%), absolute ethyl alcohol (CH_3_CH_2_OH, 99.9%, Fermont, Monterrey, Nuevo León, Mexico), monohydrate citric acid (C_6_H_8_O_7_, 99%, Avantor-J.T. Baker, Center Valley, PA, USA), hydrochloric acid (HCl), physiological saline solution, culture medium RPMI 1640 (Sigma-Aldrich, St. Louis, MO, USA), phytohemagglutinin M (Gibco, Grand Island, NY, USA), mitomycin C (C_15_H_18_N_4_O_5_, Sigma-Aldrich, St. Louis, MO, USA), cytochalasin β (C_29_H_37_NO_5_, Sigma-Aldrich, St. Louis, MO, USA), and potassium chloride (KCl, J.T. Baker, Center Valley, PA, USA).

### 4.2. Aerogel Synthesis Method

The samples were produced using a sol–gel process with an epoxide-assisted gelling method [8,36]. First, 0.1 g of scandium oxide was mixed with europium oxide at various concentrations (Sc:Eu = 100:0, 98:2, 92:8, 90:10, 70:30, and 50:50). Then, 1 mL of hydrochloric acid (HCl) was added, and the mixture was stirred continuously until the metal oxides were converted into metal salts, as shown in Equation (3).

(3) $$Sc_{2}O_{3}+6\mathrm{HCl}\to 2{\mathrm{ScCl}}_{3}+3H_{2}O$$

**Figure 10:**
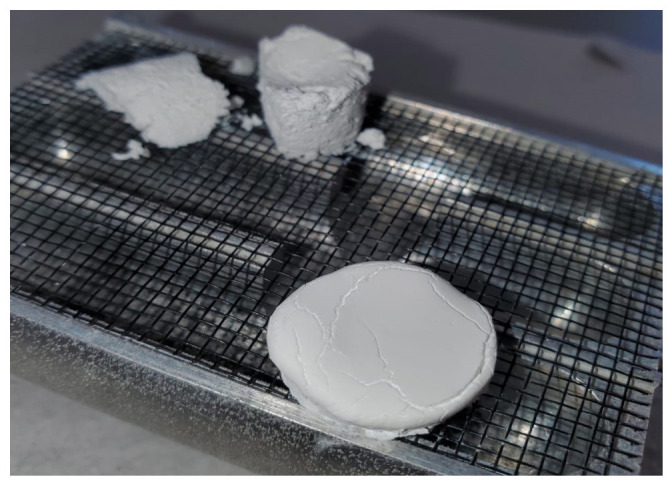
Sc_2_O_3_ aerogels with the 2% concentration of Eu^3+^.

Once this solution was obtained, the solvent was added with vigorous stirring to ensure complete homogenization and formation of the sol. Subsequently, epoxide and chelating agents were added under vigorous agitation to obtain alcoholic gels, followed by heat treatment at 700 °C for 24 h. Finally, supercritical drying was carried out at 74 bar, and the ceramic powders and aerogels were heat-treated at 35 °C to yield the aerogel as shown in Figure 10.

### 4.3. Testing Strategy

After synthesizing the products, their structural and morphological properties and DNA damage were characterized using XRD to determine the crystalline structures and sizes of the crystallites of the particles and the influence that they have. SEM and TEM were performed to determine the morphology, the shapes of the surface structures, and the interplanar distances of the nanoparticles, which were then subjected to cytogenetic testing to determine their toxicity. The lymphocyte cytokinesis-block micronucleus cytome assay (CBMC) was employed as an in vitro measure of chromosome breaks, following the Fenech methodology [37,38,39,40], to evaluate the genotoxic and cytotoxic actions of the particles on the cells.

Applying the exclusion criteria established by Fenech [22], three donors were selected to give blood for the cytogenetic assays. For each assay, 5 mL of RPMI 1640 medium was inoculated with 0.15 mL of phytohemagglutinin M and peripheral blood to stimulate cell division and proliferation.

Before culturing the cells, all tubes were incubated at 37 °C for 72 h and gently shaken twice a day to homogenize the medium and resuspend the blood cells. After 24 h, the xenobiotic agents were added to the tubes, and the scandium particles (Sc_2_O_3_:Eu_2_O_3_ at 2%) were dispersed in saline solution to prepare a stock with the concentrations shown in Table 1. In addition, 400 µg mL^−1^ of mitomycin C was added as a positive control to evaluate cytostasis, genotoxicity, and cytotoxicity in comparison with cells exposed to the nanoparticles. As a final step, before culturing the cells, 4.5 µg mL^−1^ of cytochalasin β was added to stop cellular proliferation during the last phase of division.

At 72 h of cell culture, 8 mL of prewarmed (37 °C) hypotonic 0.075 M KCl solution was added to the tubes and gently mixed to allow the cells to swell for 1 h in a water bath. Then all the tubes were put into a spin cycle for 10 min at 1500 rpm to eliminate the supernatant, leaving less than 1 mL of globular package on the tubes.

**Figure 11:**
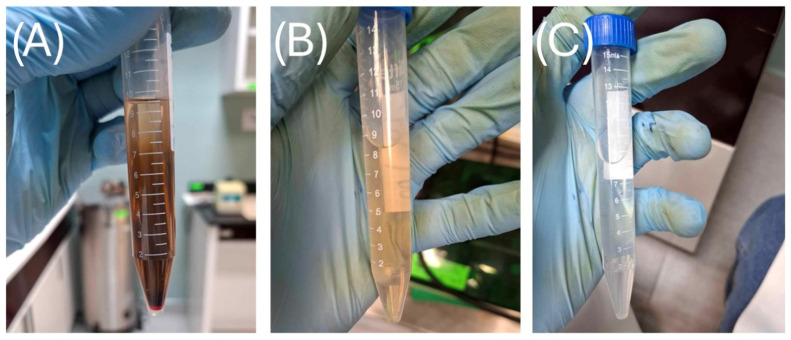
Cellular culture of lymphocytes with Carnoy’s fixative (a mixture of methanol and acetic acid at a 1:3 concentration) across (**A**) the first wash, (**B**) the second wash and (**C**) the third wash.

Once the tubes were removed from the water bath, another ten-minute spin at 1500 rpm was performed to remove the supernatant, and the globular package was resuspended using a vortex at low speed. Then three washes with Carnoy’s fixative solution were performed. For the first wash, 8 mL of Carnoy’s fixative (1:3) was added dropwise; after the first ten drops, the remaining solution was added while carefully controlling the vortex speed, and the suspension gradually became lighter. The tubes were then stored at 4 °C for 10 min to promote erythrocyte lysis. These steps were repeated for the second and third washes; however, after the first wash, the fixative was added all at once rather than dropwise until the suspension became completely transparent, leaving only the lymphocytes, as shown in Figure 11.

Finally, after the third wash and the removal of the supernatant, less than 0.5 mL of the suspension containing the lymphocytes remained, and three to four drops were deposited onto a microscope slide. Afterward, the slides were stained with Giemsa to highlight chromosomal and structural anomalies.

## Figures and Tables

**Table 1:** Experimental groups used in the cytogenetic assays of the Sc_2_O_3_:Eu_2_O_3_ system (where T1 and T2 represent the ceramic powders and the aerogels, respectively), and the concentrations used for the CBMC.

Experimental Groups	T1 (Ceramic Powders) Sc_2_O_3_:Eu_2_O_3_ (2%)	T2 (Aerogels) Sc_2_O_3_:Eu_2_O_3_ (2%)
Negative control	Saline solution (1.38 µL)	Saline solution (1.38 µL)
Positive control	Mitomycin (400 µg mL^−1^)	Mitomycin (400 µg mL^−1^)
Concentration 1	1 µg mL^−1^	1 µg mL^−1^
Concentration 2	0.1 µg mL^−1^	0.1 µg mL^−1^
Concentration 3	0.01 µg mL^−1^	0.01 µg mL^−1^

## Data Availability

Due to the complexity of the methodology and the specialized nature of the data, the full dataset supporting the findings of this study is not included in the manuscript but is available from the corresponding author upon reasonable request.

## Abbreviations

The following abbreviations are used in this manuscript:

CBMC	Cytokinesis-block micronucleus cytome assay
MNi	Micronuclei
NPBs	Nucleoplasmic bridges
NBUDs	Nuclear buds
XRD	X-ray diffraction
CRSS	Critical resolved shear stress
SEM	Scanning electron microscopy
HRTEM	High-resolution transmission electron microscopy
T1	Ceramic powder treatment
T2	Aerogel treatment
NDI	Nuclear division index
ROS	Reactive oxygen species
HCl	Hydrochloric acid
